# Preparation of a neutral nitrogen allotrope hexanitrogen *C*_2h_-N_6 _

**DOI:** 10.1038/s41586-025-09032-9

**Published:** 2025-06-11

**Authors:** Weiyu Qian, Artur Mardyukov, Peter R. Schreiner

**Affiliations:** Institute of Organic Chemistry, Justus Liebig University Giessen, Giessen, Germany

**Keywords:** Chemical bonding, Chemical synthesis, Materials chemistry

## Abstract

Compounds consisting only of the element nitrogen (polynitrogens or nitrogen allotropes) are considered promising clean energy-storage materials owing to their immense energy content that is much higher than hydrogen, ammonia or hydrazine, which are in common use, and because they release only harmless nitrogen on decomposition^1^. However, their extreme instability poses a substantial synthetic challenge and no neutral molecular nitrogen allotrope beyond N_2_ has been isolated^2,3^. Here we present the room-temperature preparation of molecular N_6_ (hexanitrogen) through the gas-phase reaction of chlorine or bromine with silver azide, followed by trapping in argon matrices at 10 K. We also prepared neat N_6_ as a film at liquid nitrogen temperature (77 K), further indicating its stability. Infrared and ultraviolet–visible (UV-Vis) spectroscopy, ^15^N-isotope labelling and ab initio computations firmly support our findings. The preparation of a metastable molecular nitrogen allotrope beyond N_2_ contributes to our fundamental scientific knowledge and possibly opens new opportunities for future energy-storage concepts.

## Abstract (web summary)

The facile synthesis and spectroscopic identification of previously unreported hexanitrogen *C*_2h_-N_6_ is described, representing experimentally realized neutral molecular nitrogen allotropes beyond N_2_ that exhibit unexpected stability.

## Main

**Fig. 1:**
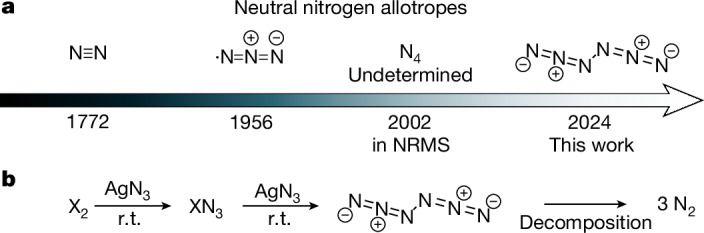
All known neutral molecular nitrogen allotropes and preparation of N_6_. **a**, Discovery timeline (year given), composition and structure (the structure of N_4_ has not been determined). **b**, Reaction sequence used in this study. r.t., room temperature.

Molecular nitrogen allotropes beyond N_2_ are promising for the development of high-energy-density materials^4^ because they release enormous energy on dissociation into gaseous N_2_. As the main component of air, N_2_ is inert, non-toxic and not a greenhouse contributor^5–7^. Unlike carbon, N_2_ is the only nitrogen allotrope found in nature and strategies for synthesizing higher neutral molecular nitrogen allotropes are highly sought after^8–14^. However, they are deemed extremely unstable, especially when uncharged and with an even electron count^15^. Consequently, only two examples have been reported. The azide radical (•N_3_) (Fig. 1a) was identified in the gas phase through rotational spectroscopy in 1956 (refs. ^16,17^). In 2002, N_4_ was detected by gas-phase neutralization-reionization mass spectrometry (NRMS); its structure has not been revealed^18^. The intermediacy of an N_6_ species was tentatively suggested in 1970 in the decay of azide radicals in aqueous solution but no definitive spectroscopic evidence was provided^19^.

There are many computations proposing molecular allotropes spanning from N_4_ to N_120_, including chains, rings and cages^7,20^, most of which have low dissociation barriers into N_2_. For example, hexazine (*cyc*-N_6_, the nitrogen analogue of benzene) exhibits a computed barrier of only 4.2 kcal mol^−1^ for decomposition into three N_2_ (ref. ^21^). Quantum mechanical tunnelling (QMT) effects could further reduce the lifetime of higher nitrogen allotropes, adding to their difficulty of preparation^22^.

Although the pursuit of higher neutral molecular nitrogen allotropes is extremely challenging, several homonuclear polynitrogen ions have been isolated. The synthesis and characterization of [N_5_]^+^[PnF_6_]^−^ (Pn = As, Sb) salts with a bent pentanitrogen cation represents a milestone^23,24^. Christe et al. initially identified the $cyclo{{\rm{-N}}}_{5}^{-}$ anion using mass spectrometry in 2002 and 2003 (refs. ^25,26^) and Zheng et al. reported in 2017 the synthesis of a salt featuring the $cyclo{{\rm{-N}}}_{5}^{-}$ anion^27^. The synthesis of various metal pentazolates was achieved through the reaction of [Na(H_2_O)(N_5_)]•2H_2_O with metal salts^28,29^.

In the realm of solid-state (non-molecular) structures, a breakthrough was the high-temperature (2,000 K), high-pressure (110 GPa) diamond-like solid-state cubic gauche nitrogen phase in which all atoms are connected by single bonds^30,31^. An aromatic cyclic hexazine ${{\rm{N}}}_{6}^{4-}$ was identified through solid-state X-ray diffraction of K_9_N_56_ under pressures above 40 GPa and temperatures above 2,000 K (ref. ^32^). Greschner et al. predicted a new nitrogen molecular crystal comprising N_6_ units with an open-chain structure stabilized by electrostatic interactions^33^, in line with assessments for the molecular species^9^.

In our analysis of the proposed molecular nitrogen allotropes, acyclic neutral N_6_ (hexaaza-1,2,4,5-tetraene, hexanitrogen, diazide) stands out because N_2_ moieties are not discernible (Fig. 1). The central N–N bond would lead to unproductive endothermic dissociation (Δ*G*_298K_ theor. +26.1 kcal mol^−1^, vide infra) into two •N_3_. Furthermore, the computed dissociation barrier into three N_2_ molecules of Δ*G*^‡^_298K_ = 14.8 kcal mol^−1^ makes N_6_ a promising candidate for synthesis. Here we show that N_6_ indeed can be prepared at room temperature through the reaction of Cl_2_ or Br_2_ with AgN_3_ under reduced pressure, followed by cryogenic trapping^34^. The characterization was accomplished by infrared (including ^15^N-isotope labelling) as well as UV-Vis spectroscopy and ab initio computations. We also demonstrate the preparation and stability of *C*_2h_-symmetric N_6_ (hereinafter referred to as N_6_) in neat form as a film at the temperature of liquid nitrogen (77 K).

## Synthesis of N_6_

**Fig. 2:**
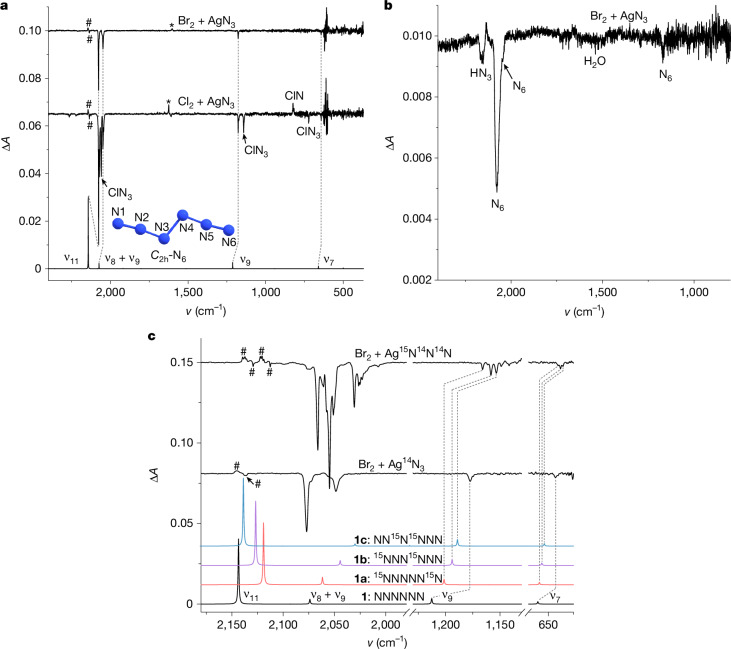
Infrared spectra of N_6_ isotopomers and side products. **a**, Lower trace: computed anharmonic infrared spectrum of N_6_ at B3LYP/def2-TZVP, including the ν_8_ + ν_9_ combination. Middle trace: difference spectrum showing the changes after 8 min of 436 nm irradiation of the products of the reaction of Cl_2_ with AgN_3_. Upper trace: difference spectrum showing the changes after 6 min of 436 nm irradiation of the reaction products of Br_2_ with AgN_3_. **b**, Difference spectrum of a neat N_6_ film at 77 K showing the changes after 8 min of 436 nm irradiation. **c**, Bottom to top traces: computed anharmonic infrared spectrum of N_6_, ^15^NNNNN^15^N (**1a**), ^15^NNN^15^NNN (**1b**) and NN^15^N^15^NNN (**1c**) at B3LYP/def2-TZVP, including the ν_8_ + ν_9_ combination; difference spectrum showing the changes after 8 min of 436 nm irradiation of the reaction products of Br_2_ with AgN_3_; difference spectrum showing changes after 8 min of 436 nm irradiation of the reaction products of Br_2_ with Ag^15^N^14^N^14^N. Matrix sites from natural abundance and isotope-labelled HN_3_ (#) and H_2_O (*) are marked.

As AgN_3_ is an excellent reagent for the synthesis of polyazides^35^ and halogen azides both in the gas phase^36^ and in solution^37,38^, we suggest that the reaction of AgN_3_ with XN_3_ (X = halogen) is a viable route to N_6_ (Fig. 1b). The reactions were conducted in either a quartz tube or a U-trap by flowing gaseous Cl_2_ through solid AgN_3_ under reduced pressure at room temperature (see the ‘Synthesis details’ section in Methods and Supplementary Fig. 1). Apart from the known bands of ClN_3_ (ref. ^39^) and HN_3_ (ref. ^40^), a distinct group of bands at 2,076.6, 2,049.0, 1,177.6 and 642.1 cm^−1^ was recorded (Supplementary Fig. 2). After irradiating the matrices with 436 nm light (Fig. 2a middle trace and Supplementary Fig. 3), all bands vanish. However, the rates of decomposition of the newly observed infrared bands differ from those attributed to ClN_3_ (Supplementary Figs. 4 and 5). There were no discernible products other than chloronitrene (ClN) detected in the difference spectrum after irradiation. Furthermore, identical bands were detected when Br_2_ was used instead of Cl_2_, indicating that the unidentified species does not contain halogens (Fig. 2a upper trace and Supplementary Fig. 6). Also, BrN_3_ does not decompose on 436 nm irradiation, providing clean decomposition spectra of the yet unidentified species.

The intensive vibrational band at 2,076.6 cm^−1^ compares favourably with the asymmetric stretching band of the azide moiety in isoelectronic N_3_–NCO (2,099.1 cm^−1^, Ar matrix)^41^. Compared with the computed harmonic vibrations at CCSD(T)/cc-pVTZ, the four bands noted above could be attributed to N_6_, except the band at 2,049.0 cm^−1^ of moderate intensity, although it disappeared together with the other bands following photolysis (Supplementary Figs. 4 and 7). To determine the origin of the band at 2,049.0 cm^−1^, anharmonic vibrational frequencies were computed at B3LYP/def2-TZVP (Supplementary Table 1). This analysis indicates that this band derives from a combination of fundamentals ν_8_ (a_*g*_ symmetric N^3^N^4^ stretching mode) and ν_9_ (b_*u*_ asymmetric N^3^N^2^N^1^ stretching mode). The substantial anharmonic intensity contribution (219 km mol^−1^; Supplementary Table 2) of the fundamental ν_11_ at 2,143.5 cm^−1^ and the ν_8_ + ν_9_ combination is notably stronger than its fundamentals, suggesting that the combination ν_8_ + ν_9_ gains energy through Fermi resonance from the adjacent strong fundamental ν_11_ (ref. ^41^).

**Fig. 3:**
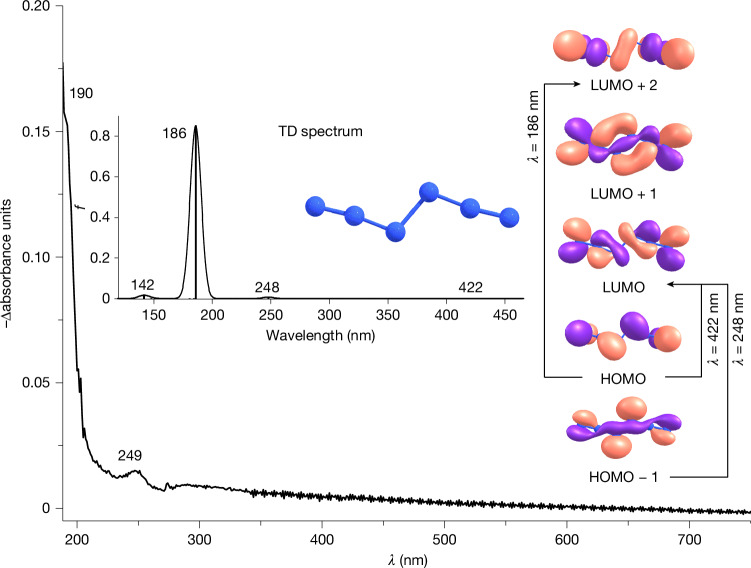
Measured and computed UV-Vis spectrum of N_6_ and molecular orbitals involved in the electronic transitions. Experimental difference UV-Vis spectrum reflecting changes following 4 min of 436 nm irradiation of the reaction products of Br_2_ with AgN_3_ in argon at 10 K. Inset, computed [TD-B3LYP/def2-TZVP] electronic transitions for N_6_ and molecular orbitals involved.

To confirm our assignments, isotope-labelling experiments were conducted using Ag^15^N^14^N^14^N. Three groups of distinct peaks can be discerned in the infrared spectra (Fig. 2c and Supplementary Fig. 8), indicating the presence of two N_3_ moieties in the molecule, which can be attributed to three types of isotopomer (**1a**: ^15^NNNNN^15^N, **1b**: ^15^NNN^15^NNN, **1c**: NN^15^N^15^NNN), respectively. In particular, the unsymmetric isotopic substitutions in **1b** lower its point group from *C*_2h_ to *C*_s_. Computations delineate that the terminal (N^1^ or N^6^) and internal (N^3^ or N^4^) ^15^N substitutions mainly influence the terminal (ν_11_) and internal asymmetric stretching vibration (ν_9_) of the N_3_ moieties, respectively. This leads to a redshift of the ν_8_ + ν_9_ combination and a blueshift of the ν_11_ fundamental in going from **1a** to **1c**, resulting in their gradual separation. The intensity ratio of the ν_8_ + ν_9_ combination and the ν_11_ fundamental in **1a** is nearly 1:1, which is much higher than that in **1c** (about 1:17). These findings align well with the anharmonic infrared intensities computed by density functional theory (Supplementary Table 3), which are attributed to the closer proximity of the ν_8_ + ν_9_ combination to the strong ν_11_ fundamental in **1a**, resulting in an increase of the Fermi resonance and vice versa in **1c**. Statistically, the anticipated ratio of the three isotopomers should be **1a**:**1b**:**1c** = 1:2:1, which is reflected in the observed fundamental ν_7_ in the experimental spectrum (Fig. 3). Furthermore, the computed intensity of ν_9_ in **1c** (107 km mol^−1^; Supplementary Table 3) is higher than that in **1a** (92 km mol^−1^) and **1b** (98 km mol^−1^), which matches the intensity ratios of ν_9_ observed in **1a** and **1b** (approximately 1:2). The experimentally observed intensities agree with these findings and show a slightly higher intensity of ν_9_ in **1c** than in **1a**.

To explore the intrinsic stability of N_6_, we also prepared neat N_6_ at room temperature and condensed it at liquid nitrogen temperature (77 K) as a film on the surface of the matrix window without using argon as a host gas. Irradiation of such N_6_ films resulted in very similar spectral changes as those observed in argon matrices at 10 K (Fig. 2b and Supplementary Fig. 9). That is, neat N_6_ is sufficiently stable at the temperature of liquid nitrogen to allow its direct identification.

Further evidence is provided by the UV-Vis spectrum of N_6_. After 6 min of 436 nm irradiation of the reaction products of Br_2_ with AgN_3_, we observed the disappearance of the transitions at 190 and 249 nm and, consistent with the infrared experiments, no new transitions appeared (Fig. 3). All transitions correlate well with the values for the electronic excitations of N_6_ at 186 nm (*f* = 0.8512) and 248 nm (*f* = 0.0078) computed at [TD-B3LYP/def2-TZVP]. Furthermore, the computations reveal a weak electronic excitation at 422 nm (*f* = 0.0004), corresponding to a π → π* transition, which aligns well with the observed photochemistry.

## Computations

**Fig. 4:**
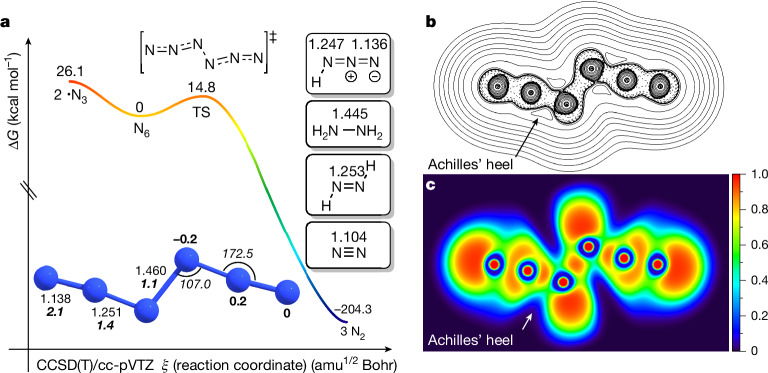
Computational analyses for N_6_. **a**, Potential energy profile (Δ*G*_298K_, kcal mol^−1^) for N_6_ at CCSD(T)/cc-pVTZ. The optimized parameters of N_6_ are given in Ångstrom (normal font), degrees (italics), natural charges in bold and natural bond orders in bold italics. Insets, computed NN bond lengths for N_2_, *trans*-HNNH, hydrazine and HN_3_ at CCSD(T)/cc-pVTZ. **b**, Contour line map of the Laplacian of the electron density of N_6_; solid and dashed lines represent positive and negative regions, respectively. **c**, ELF map.

To better understand the structure and the potential energy landscape of N_6_, we computed its energy profile at CCSD(T)/cc-pVTZ (Fig. 4a (Δ*G*_298K_) and Supplementary Fig. 10 (Δ*H*_0_); see the ‘Computational details’ section in Methods). Only the *C*_2h_-N_6_ *trans*-conformer is a local minimum; the *C*_2v_-N_6_ *cis*-conformer is a higher-order stationary point and chemically not relevant^42,43^. The formal double bond lengths in the N_3_ moieties are much longer than the triple bond in N_2_ (theor. 1.104 Å; expt. 1.098 Å)^44^, indicating double-bond character. Indeed, the computed N^2^ = N^3^/N^4^ = N^5^ bond length (1.251 Å) is close to that of *trans*-diazene (HN = NH, theor. 1.253 Å; expt. 1.252 Å)^45^. The structure of N_6_ is different from the azide radical (•N = N = N, theor. 1.183 Å; expt. 1.181 Å)^46^ but comparable with the N_3_ moiety in hydrazoic acid (HN_3_, theor. 1.247 and 1.136 Å; expt. 1.237 and 1.133 Å for the N^1^ = N^2^ and N^2^ = N^3^ bonds, respectively). The N^3^–N^4^ bond in N_6_ (1.460 Å) compares favourably with that in hydrazine (H_2_N–NH_2_, theor. 1.445 Å; expt. 1.446 Å). This geometric analysis is well captured by the Lewis structure of N_6_ (Fig. 1). These conclusions are supported by natural bond orbital computations, which indicate that the terminal nitrogen atoms are electronically neutral, whereas small positive and negative charges are located at N^2^ and N^5^ (+0.2*e*) as well as on N^3^ and N^4^ (−0.2*e*), respectively (Fig. 4a). Equally, N^1^–N^2^/N^5^-N^6^ have the highest bond order (2.1), followed by N^2^–N^3^/N^4^–N^5^ (1.4) and N^3^–N^4^ (1.1).

We visualized the Laplacian of the electron density to gauge where the bonds in N_6_ are likely to break (Fig. 4b) and why the computed barrier for decomposition into three moles of N_2_ is, compared with other systems, rather high (Δ*G*^‡^_298K_ = 14.8 kcal mol^−1^). This barrier implies appreciable kinetic stability that is mirrored by our observations. For comparison, the computed barrier of hypothetical *D*_2h_-N_4_ dissociating into two N_2_ is 6.5 kcal mol^−1^ at MR-AQCC/VTZ^47^. With the electron density analysis, the ‘Achilles’ heel’ was discerned at the N^2^–N^3^/N^4^–N^5^ bonds, as evident from the vertex of positive Laplacian of the in-plane electron density. This is confirmed by the electron localization function (ELF) analysis^48^ (Fig. 4c). Both the Laplacian of the electron density and the ELF analysis indicate the electron density minimum around the N^2^–N^3^/N^4^–N^5^ bonds. Hence, even though the Lewis structure would indicate N_6_ breaking into two •N_3_ radicals, that is, breaking of the central N^3^–N^4^ single bond, the computed barrier for this process amounts to sizeable Δ*G*_298K_ = 26.1 kcal mol^−1^ and is unproductive.

On the other hand, Δ*G*^‡^_298K_ for the elementary decomposition into three N_2_ is 14.8 kcal mol^−1^, implying a finite lifetime of N_6_ at room temperature. As N_6_ decomposition may be accelerated by QMT^21,22,49^, we used canonical variational theory and small-curvature tunnelling computations at B3LYP/def2-TZVP that reveal that N_6_, unlike hexazine (*cyc*-N_6_)^21^, is unlikely to decompose through QMT, with an estimated half-life of N_6_ of more than 132 years at 77 K (Supplementary Table 4). At 298 K, the computed half-life still amounts to 35.7 ms. This supports our finding that N_6_ exists long enough in the gas phase at ambient temperature to be trapped subsequently in cryogenic matrices.

According to CCSD(T)/cc-pVTZ (Δ*H*_0_) computations, the decomposition of N_6_ into three N_2_ is exothermic (Δ*H*_0_) by 185.2 kcal mol^−1^, which is 2.2 and 1.9 times higher than the decomposition enthalpies of TNT (2,4,6-trinitrotoluene) and HMX (1,3,5,7-tetranitro-1,3,5,7-tetrazocane, octogen) by weight^50^ (see the ‘Computational details’ section in Methods).

We report here the facile synthesis and spectroscopic identification of experimentally unreported hexanitrogen N_6_. This represents the first, to our knowledge, experimentally realized neutral molecular nitrogen allotrope beyond N_2_ that exhibits unexpected stability. This discovery challenges the long-held belief of the elusiveness of neutral molecular nitrogen allotropes.

## Methods

### Matrix apparatus design

For the matrix isolation studies, we used an APD Cryogenics HC-2 cryostat with a closed-cycle refrigerator system, equipped with an inner CsI window for infrared measurements. Spectra were recorded at the temperature of the matrix (10 K) with a Bruker VERTEX 70 FT-IR spectrometer with a spectral range of 4,000–400 cm^−1^ and a resolution of 0.7 cm^−1^ and UV-Vis spectra were recorded with a Jasco V-670 spectrophotometer equipped with an inner sapphire window. A high-pressure mercury lamp (HBO 200, Osram) with a monochromator (Bausch & Lomb) was used for irradiation. Cl_2_ or Br_2_ was evaporated (Cl_2_-CCl_4_: −140 °C, Br_2_: −85 °C) from a storage bulb into the quartz tube or U-trap. Although not directly measured, all reaction products were co-condensed with a large excess of argon (typically 60–120 mbar from a 2,000-ml storage bulb) onto the surface of the matrix window at 10 K in several milliseconds.

### Synthesis details

Warning! Silver azide and halogen azides are extremely hazardous and explosive. Such compounds should be handled with utmost care and only in very small quantities (<5 mmol). Appropriate safety precautions (blast screens, face shields, Kevlar gloves, soundproof earmuffs and protective leather clothing) are necessary. Make sure to eliminate static electricity before handling. It is also crucial to avoid friction and light exposure and prevent any contact with metals during sample handling to ensure safety.

Silver azide was synthesized by adding a stoichiometric amount of a silver nitrate–water solution to a sodium azide–water solution in the dark. The precipitate was washed three times with anhydrous ethanol. The resulting slurry was loosely dispersed on one side of the inner surface of a straight quartz tube (ø 10 × 1) or the inner surface of a U-trap (inside diameter 10 mm) and then brought to reduced pressure to remove the solvent. Typically, 0.6 mmol and 2.5 mmol of AgN_3_ are required for the straight quartz tube and U-trap, respectively. Na^15^N^14^N^14^N (>99% ^15^N, Sigma-Aldridge) was used for isotope labelling experiments. Chlorine gas was bubbled into CCl_4_ at 0 °C and degassed before use. Bromine was purified by vacuum distillation before use. Typically, 3 mmol of halogen were stored in the storage bulb for the reaction.

### Computational details

Geometry optimizations and energy computations were carried out at the CCSD(T)/cc-pVTZ^51–53^ levels of theory using ORCA 5.0 (with keywords verytightscf and verytightopt)^54^. B3LYP^55,56^ computations (geometry optimizations, energy computations (all free energies were computed at 298 K), harmonic vibrational analysis and DVPT2 anharmonic vibrational analysis) were performed using Gaussian 16 (ref. ^57^) with a def2-TZVP basis set^58^. Local minima were confirmed by vibrational frequencies analyses and transition states were further confirmed by intrinsic reaction coordinate computations. Harmonic vibrational analysis at CCSD(T)/cc-pVTZ was performed using CFOUR v2.1 (ref. ^59^). Wavefunction analysis (Laplacian of electron density and electron localization function) results were obtained from Multiwfn 3.8 (ref. ^60^) at CCSD(T)/cc-pVTZ. Natural bond order analysis and resonance structures were computed with NBO 7.0 (refs. ^61,62^). CVT/SCT (canonical variational transition state theory with small-curvature tunnelling) and CVT/ZCT (canonical variational transition state theory with zero-curvature tunnelling) computations were carried out with Gaussrate 17 (refs. ^27,63–67^) as an interface between Gaussian 16 and Polyrate^68^. Furthermore, local stretching force constants were obtained by LModeA-nano^69^ as a plugin of the open-source version of the visualization program PyMOL.

### Detonation calculation details

First, the density (*ρ*, in cm^3^ per molecule) of the N_6_ crystal was determined using electrostatic interaction correction as suggested by Politzer et al.^70^ (equation (2)). *M*_m_ (84.04/(6.02 × 10^23^) g per molecule) is the molecular mass. *V*_m_ (610.52/(1.89 × 10^8^)^3^ cm^3^ per molecule) is the volume of the isolated gas-phase molecule, which was determined by the 0.001 a.u. density envelope using the marching tetrahedron method^60,71^. *ν* is the parameter of balance between positive and negative surface potentials^72^ (equation (2)). ${\sigma }_{{\rm{tot}}}^{2}$ (48.40 kcal^2^ mol^−2^) is the strengths and variabilities of the overall surface potentials, which could be derived from variance of positive (${\sigma }_{+}^{2}$, 31.06 kcal^2^ mol^−2^) and negative charges (${\sigma }_{-}^{2}$, 17.34 kcal^2^ mol^−2^) with equation (3). *α* (0.9183), *β* (0.0028) and *γ* (0.0443) are coefficients.

1 $$\rho =\alpha \left(\frac{{M}_{{\rm{m}}}}{{V}_{{\rm{m}}}}\right)+\beta (\nu {\sigma }_{{\rm{tot}}}^{2})+\gamma $$

2 $$\nu =\frac{{\sigma }_{+}^{2}{\sigma }_{-}^{2}}{{({\sigma }_{+}^{2}+{\sigma }_{-}^{2})}^{2}}$$

3 $${\sigma }_{{\rm{tot}}}^{2}={\sigma }_{+}^{2}+{\sigma }_{-}^{2}$$

The detonation velocity (*D*) and detonation pressure (*P*) were calculated using the Kamlet–Jacobs equation^73^ (equations (4) and (5)). *N* is the number of moles of the gas generated per gram (equation (6)), $\bar{M}$ is the average molecular weight of the gaseous product (equation (7)), *Q* is the heat of detonation (equation (8)), *M* is the molecular weight (84.04 g mol^−1^), Δ*H*_f_ is the standard heat of formation (774.88 kJ mol^−1^, which was derived from the energy difference of the computed enthalpy at 298 K between *C*_2h_-N_6_ and 3 moles of N_2_) and *a* (0), *b* (0), *c* (0) and *d* (6) represent the number of C, H, O, and N atoms in the molecule, respectively.

4 $$D=1.01{(N\sqrt{\bar{M}Q})}^{\frac{1}{2}}(1+1.3\rho )$$

5 $$P=1.558{\rho }^{2}N\sqrt{\bar{M}Q}$$

6 $$N=\frac{b+2c+2d}{4M}$$

7 $$\bar{M}=\frac{4M}{b+2c+2d}$$

8 $$Q=\frac{28.9b+94.05a+0.239\Delta {H}_{{\rm{f}}}}{M}$$

Assessing the energetic performance using the Kamlet–Jacobs equation^73^, the CCSD(T)/cc-pVTZ level of theory predicts a lower density (*ρ*: 1.51 g cm^−3^) than that of TNT (1.65 g cm^−3^) and an excellent detonation performance (detonation velocity *D*: 8,930 m s^−1^; detonation pressure *P*: 31.7 GPa). This compares favourably with several well-known explosives, for example, TNT (*D*: 6,900 m s^−1^; *P*: 21.0 GPa), RDX (1,3,5-trinitro-1,3,5-triazinane; *D*: 8,750 m s^−1^; *P*: 34.5 GPa) and FOX-7 (1,1-diamino-2,2-dinitroethylene; *D*: 8,870 m s^−1^; *P*: 34.5 GPa)^74^.

### Energy-releasing equivalent calculation details

A kiloton of N_6_ is 1.19 × 10^7^ mol, which can release an energy of 2.20 × 10^9^ kcal (9.21 terajoules) based on the enthalpy (Δ*H*_0_). Considering that the standard kiloton TNT equivalent is 4.184 terajoules, N_6_ can release 2.2 times the energy of TNT of the same weight. On the basis of the documented TNT equivalent based on weight for HMX (1.15) and RDX (1.15)^50^, N_6_ can release 1.9 times the energy of HMX or RDX with the same weight.

## Online content

Any methods, additional references, Nature Portfolio reporting summaries, source data, extended data, supplementary information, acknowledgements, peer review information; details of author contributions and competing interests; and statements of data and code availability are available at 10.1038/s41586-025-09032-9.

## Supplementary information

Supplementary Information This file contains a note for the synthesis, Supplementary Figs. 1–15, Supplementary Tables 1–4 as well as xyz-coordinates of all computed species.

## References

[1] Christe, K. O. Polynitrogen chemistry enters the ring. *Science* **355**, 351–351 (2017). 28126772 10.1126/science.aal5057

[2] Wang, Y. et al. Stabilization of hexazine rings in potassium polynitride at high pressure. *Nat. Chem.* **14**, 794–800 (2022). 35449217 10.1038/s41557-022-00925-0

[3] Ninet, S. Benzene-like N_6_ hexazine rings. *Nat. Chem.* **15**, 595–596 (2023). 37095405 10.1038/s41557-023-01189-y

[4] Yao, Y. & Adeniyi, A. O. Solid nitrogen and nitrogen‐rich compounds as high‐energy‐density materials. *Phys. Status Solidi B* **258**, 2000588 (2021).

[5] Klapötke, T. M. & Witkowski, T. G. Nitrogen-rich energetic 1,2,5-oxadiazole-tetrazole-based energetic materials. *Propellants Explos. Pyrotech.* **40**, 366–373 (2015).

[6] Nguyen, M. T. Polynitrogen compounds: 1. Structure and stability of N_4_ and N_5_ systems. *Coord. Chem. Rev.* **244**, 93–113 (2003).

[7] Zarko, V. E. Searching for ways to create energetic materials based on polynitrogen compounds (review). *Combust. Explos. Shock Waves* **46**, 121–131 (2010).

[8] Larson, Å., Larsson, M. & Östmark, H. Theoretical study of rectangular (*D*_2h_) N_4_. *J. Chem. Soc. Faraday Trans.* **93**, 2963–2966 (1997).

[9] Glukhovtsev, M. N. & von Ragué Schleyer, P. Structures, bonding and energies of N_6_ isomers. *Chem. Phys. Lett.* **198**, 547–554 (1992).

[10] Glukhovtsev, M. N., Jiao, H. & von Ragué Schleyer, P. Besides N_2_, what is the most stable molecule composed only of nitrogen atoms? *Inorg. Chem.* **35**, 7124–7133 (1996). 11666896 10.1021/ic9606237

[11] Strout, D. L. Acyclic N_10_ fails as a high energy density material. *J. Phys. Chem. A* **106**, 816–818 (2002).

[12] Hirshberg, B., Gerber, R. B. & Krylov, A. I. Calculations predict a stable molecular crystal of N_8_. *Nat. Chem.* **6**, 52–56 (2014). 24345947 10.1038/nchem.1818

[13] Strout, D. L. Cage isomers of N_14_ and N_16_: nitrogen molecules that are not a multiple of six. *J. Phys. Chem. A* **108**, 10911–10916 (2004).

[14] Samartzis, P. C. & Wodtke, A. M. All-nitrogen chemistry: how far are we from N_60_? *Int. Rev. Phys. Chem.* **25**, 527–552 (2010).

[15] Mikhailov, O. V. Molecular and electronic structures of neutral polynitrogens: review on the theory and experiment in 21st century. *Int. J. Mol. Sci.* **23**, 2841 (2022). 35269983 10.3390/ijms23052841 PMC8911370

[16] Thrush, B. A. & Norrish, R. G. W. The detection of free radicals in the high intensity photolysis of hydrogen azide. *Proc. R. Soc. Lond. A* **235**, 143–147 (1956).

[17] Beaman, R. A., Nelson, T., Richards, D. S. & Setser, D. W. Observation of azido radical by laser-induced fluorescence. *J. Phys. Chem.* **91**, 6090–6092 (1987).

[18] Cacace, F., de Petris, G. & Troiani, A. Experimental detection of tetranitrogen. *Science* **295**, 480–481 (2002). 11799238 10.1126/science.1067681

[19] Hayon, E. & Simic, M. Absorption spectra and kinetics of the intermediate produced from the decay of azide radicals. *J. Am. Chem. Soc.* **92**, 7486–7487 (1970).

[20] Zhou, H., Wong, N.-B., Zhou, G. & Tian, A. Theoretical study on “multilayer” nitrogen cages. *J. Phys. Chem. A* **110**, 3845–3852 (2006). 16526671 10.1021/jp056435w

[21] Sedgi, I. & Kozuch, S. Quantum tunneling instability of the mythical hexazine and pentazine. *Chem. Commun.* **60**, 2038–2041 (2024). 10.1039/d3cc05840a 38284898

[22] Schreiner, P. R. Quantum mechanical tunneling is essential to understanding chemical reactivity. *Trends Chem.* **2**, 980–989 (2020).

[23] Christe, K. O., Wilson, W. W., Sheehy, J. A. & Boatz, J. A. N_5_^+^: a novel homoleptic polynitrogen ion as a high energy density material. *Angew. Chem. Int. Ed.* **38**, 2004–2009 (1999). 10.1002/(SICI)1521-3773(19990712)38:13/14<2004::AID-ANIE2004>3.0.CO;2-7 34182671

[24] Vij, A. et al. Polynitrogen chemistry. Synthesis, characterization, and crystal structure of surprisingly stable fluoroantimonate salts of N_5_^+^. *J. Am. Chem. Soc.* **123**, 6308–6313 (2001). 11427055 10.1021/ja010141g

[25] Vij, A., Pavlovich, J. G., Wilson, W. W., Vij, V. & Christe, K. O. Experimental detection of the pentaazacyclopentadienide (pentazolate) anion, *cyclo*-N_5_^−^. *Angew. Chem. Int. Ed.* **41**, 3051–3054 (2002). 10.1002/1521-3773(20020816)41:16<3051::AID-ANIE3051>3.0.CO;2-T 12203455

[26] Östmark, H. et al. Detection of pentazolate anion (*cyclo*-N_5_^−^) from laser ionization and decomposition of solid *p*-dimethylaminophenylpentazole. *Chem. Phys. Lett.* **379**, 539–546 (2003).

[27] Zhang, C. et al. Synthesis and characterization of the pentazolate anion cyclo-N_5_ˉ in (N_5_)_6_(H_3_O)_3_(NH_4_)_4_Cl. *Science* **355**, 374–376 (2017). 10.1126/science.aah3840 28126812

[28] Xu, Y. et al. A series of energetic metal pentazolate hydrates. *Nature* **549**, 78–81 (2017). 28847006 10.1038/nature23662

[29] Xu, Y., Tian, L., Li, D., Wang, P. & Lu, M. A series of energetic *cyclo*-pentazolate salts: rapid synthesis, characterization, and promising performance. *J. Mater. Chem. A* **7**, 12468–12479 (2019).

[30] Eremets, M. I., Gavriliuk, A. G., Trojan, I. A., Dzivenko, D. A. & Boehler, R. Single-bonded cubic form of nitrogen. *Nat. Mater.* **3**, 558–563 (2004). 15235595 10.1038/nmat1146

[31] Benchafia, E. M. et al. Cubic gauche polymeric nitrogen under ambient conditions. *Nat. Commun.* **8**, 930 (2017). 29030605 10.1038/s41467-017-01083-5 PMC5640604

[32] Laniel, D. et al. Aromatic hexazine [N_6_]^4−^ anion featured in the complex structure of the high-pressure potassium nitrogen compound K_9_N_56_. *Nat. Chem.* **15**, 641–646 (2023). 36879075 10.1038/s41557-023-01148-7

[33] Greschner, M. J. et al. A new allotrope of nitrogen as high-energy density material. *J. Phys. Chem. A* **120**, 2920–2925 (2016). 27088348 10.1021/acs.jpca.6b01655

[34] Qian, W. Y., Mardyukov, A. & Schreiner, P. R. Hexanitrogen (N_6_): a synthetic leap towards neutral nitrogen allotropes. Preprint at 10.26434/chemrxiv-2024-90vvx (2024).

[35] Zeng, X. et al. Reaction of AgN_3_ with SOCl_2_: evidence for the formation of thionyl azide, SO(N_3_)_2_. *Inorg. Chem.* **43**, 4799–4801 (2004). 15285648 10.1021/ic049442s

[36] Raschig, F. Über Chlorazid N_3_Cl. *Ber. Dtsch. Chem. Ges.* **41**, 4194–4195 (1908).

[37] Lyhs, B., Bläser, D., Wölper, C., Schulz, S. & Jansen, G. A comparison of the solid-state structures of halogen azides XN_3_ (X=Cl, Br, I). *Angew. Chem. Int. Ed.* **51**, 12859–12863 (2012). 10.1002/anie.201206028 23143850

[38] Buzek, P., Klapötke, T. M., von Ragué Schleyer, P., Tornieporth‐Oetting, I. C. & White, P. S. Iodine azide. *Angew. Chem. Int. Ed.* **32**, 275–277 (1993).

[39] Shurvell, H. F. & Hyslop, D. W. Infrared spectrum of cyanogen azide. *J. Chem. Phys.* **52**, 881–887 (1970).

[40] Pimental, G. C. & Charles, S. W. Infrared spectral perturbations in matrix experiments. *Pure Appl. Chem.* **7**, 111–124 (1963).

[41] Zeng, X., Beckers, H. & Willner, H. Matrix isolation of two isomers of N_4_CO. *Angew. Chem. Int. Ed. Engl.* **50**, 482–485 (2011). 21132825 10.1002/anie.201005177

[42] Tobita, M. & Bartlett, R. J. Structure and stability of N_6_ isomers and their spectroscopic characteristics. *J. Phys. Chem. A* **105**, 4107–4113 (2001).

[43] Gagliardi, L., Evangelisti, S., Barone, V. & Roos, B. O. On the dissociation of N_6_ into 3 N_2_ molecules. *Chem. Phys. Lett.* **320**, 518–522 (2000).

[44] Huber, K. P. & Herzberg, G. in *Molecular Spectra and Molecular Structure* (eds Huber, K. P. & Herzberg, G.) Ch. 2, 8–689 (Springer, 1979).

[45] Carlotti, M., Johns, J. W. C. & Trombetti, A. The ν_5_ fundamental bands of N_2_H_2_ and N_2_D_2_. *Can. J. Phys.* **52**, 340–344 (1974).

[46] Brazier, C. R., Bernath, P. F., Burkholder, J. B. & Howard, C. J. Fourier transform spectroscopy of the ν_3_ band of the N_3_ radical. *J. Chem. Phys.* **89**, 1762–1767 (1988).

[47] Bittererová, M., Östmark, H. & Brinck, T. Ab initio study of the ground state and the first excited state of the rectangular (D_2h_)N_4_ molecule. *Chem. Phys. Lett.* **347**, 220–228 (2001).

[48] Lu, T. & Chen, F. Multiwfn: a multifunctional wavefunction analyzer. *J. Comput. Chem.* **33**, 580–592 (2012). 22162017 10.1002/jcc.22885

[49] Schreiner, P. R. Tunneling control of chemical reactions: the third reactivity paradigm. *J. Am. Chem. Soc.* **139**, 15276–15283 (2017). 29028320 10.1021/jacs.7b06035

[50] Weggel, D. C. in *Blast Protection of Civil Infrastructures and Vehicles Using Composites* (ed. Uddin, N.) 3–43 (Woodhead Publishing, 2010).

[51] Pople, J. A., Head‐Gordon, M. & Raghavachari, K. Quadratic configuration interaction. A general technique for determining electron correlation energies. *J. Chem. Phys.* **87**, 5968–5975 (1987).

[52] Bartlett, R. J. & Purvis, G. D. Many-body perturbation theory, coupled-pair many-electron theory, and the importance of quadruple excitations for the correlation problem. *Int. J. Mol. Sci.* **14**, 561–581 (1978).

[53] Pople, J. A., Krishnan, R., Schlegel, H. B. & Binkley, J. S. Electron correlation theories and their application to the study of simple reaction potential surfaces. *Int. J. Mol. Sci.* **14**, 545–560 (1978).

[54] Neese, F. Software update: the ORCA program system—version 5.0. *WIREs Comput. Mol. Sci.* **12**, e1606 (2022).

[55] Becke, A. D. Density‐functional thermochemistry. III. The role of exact exchange. *J. Chem. Phys.* **98**, 5648–5652 (1993).

[56] Stephens, P. J., Devlin, F. J., Chabalowski, C. F. & Frisch, M. J. Ab initio calculation of vibrational absorption and circular dichroism spectra using density functional force fields. *J. Phys. Chem.* **98**, 11623–11627 (1994).

[57] Frisch, M. J. et al. Gaussian 16, Revision B.01 (Gaussian, Inc., 2016).

[58] Weigend, F. & Ahlrichs, R. Balanced basis sets of split valence, triple zeta valence and quadruple zeta valence quality for H to Rn: design and assessment of accuracy. *Phys. Chem. Chem. Phys.* **7**, 3297–3305 (2005). 16240044 10.1039/b508541a

[59] Stanton, J. F. et al. CFOUR, coupled-cluster techniques for computational chemistry, a quantum-chemical program package with the integral packages MOLECULE (J. Almlöf and PR Taylor), PROPS (PR Taylor) (2014).

[60] Lu, T. & Chen, F. Quantitative analysis of molecular surface based on improved Marching Tetrahedra algorithm. *J. Mol. Graph. Model.* **38**, 314–323 (2012). 23085170 10.1016/j.jmgm.2012.07.004

[61] Glendening, E. D., Landis, C. R. & Weinhold, F. NBO 7.0: new vistas in localized and delocalized chemical bonding theory. *J. Comput. Chem.* **40**, 2234–2241 (2019). 31172571 10.1002/jcc.25873

[62] Glendening, E. D., Landis, C. R. & Weinhold, F. Natural bond orbital methods. *WIREs Comput. Mol. Sci.* **2**, 1–42 (2012).

[63] Zheng, J. et al. *Gaussrate 17-B* (Univ. Minnesota, 2017).

[64] Garrett, B. C. & Truhlar, D. G. Generalized transition state theory. Bond energy-bond order method for canonical variational calculations with application to hydrogen atom transfer reactions. *J. Am. Chem. Soc.* **101**, 4534–4548 (1979).

[65] Garrett, B. C. & Truhlar, D. G. Criterion of minimum state density in the transition state theory of bimolecular reactions. *J. Chem. Phys.* **70**, 1593–1598 (1979).

[66] Garrett, B. C., Truhlar, D. G., Grev, R. S. & Magnuson, A. W. Improved treatment of threshold contributions in variational transition-state theory. *J. Phys. Chem.* **84**, 1730–1748 (1980).

[67] Truhlar, D. G., Issacson, A., Skodje, R. & Garrett, B. C. Additions and corrections - incorporation of quantum effects in generalized-transition-state theory. *J. Phys. Chem.* **87**, 4554–4554 (1983).

[68] Zheng, J. et al. *Polyrate-version 2017-C* (Univ. Minnesota, 2017).

[69] Tao, Y., Zou, W., Nanayakkara, S. & Kraka, E. LModeA-nano: a PyMOL plugin for calculating bond strength in solids, surfaces, and molecules via local vibrational mode analysis. *J. Chem. Theory Comput.* **18**, 1821–1837 (2022). 35192350 10.1021/acs.jctc.1c01269

[70] Politzer, P., Martinez, J., Murray, J. S., Concha, M. C. & Toro-Labbé, A. An electrostatic interaction correction for improved crystal density prediction. *Mol. Phys.* **107**, 2095–2101 (2009).

[71] Bader, R. F. W., Carroll, M. T., Cheeseman, J. R. & Chang, C. Properties of atoms in molecules: atomic volumes. *J. Am. Chem. Soc.* **109**, 7968–7979 (1987).

[72] Murray, J. S., Concha, M. C. & Politzer, P. Links between surface electrostatic potentials of energetic molecules, impact sensitivities and C–NO_2_/N–NO_2_ bond dissociation energies. *Mol. Phys.* **107**, 89–97 (2009).

[73] Kamlet, M. J. & Jacobs, S. J. Chemistry of detonations. I. A simple method for calculating detonation properties of C–H–N–O explosives. *J. Chem. Phys.* **48**, 23–35 (1968).

[74] Prazyan, T. L. & Zhuravlev, Y. N. Computer simulation of the structure and electronic and detonation properties of energy materials. *Combust. Explos. Shock Waves* **53**, 718–723 (2017).

