# Supplementary material for: Preparation of a neutral nitrogen allotrope hexanitrogen C2h-N6
Source: Nature. 2025 Jun 11;642(8067):356–60. doi: 10.1038/s41586-025-09032-9 (PMC12158757; doi:10.1038/s41586-025-09032-9)
Supplement: Supplementary file 1 — This file contains a note for the synthesis, Supplementary Figs. 1–15, Supplementary Tables 1–4 as well as xyz-coordinates of all computed species. [file 41586_2025_9032_MOESM1_ESM.pdf]

nature portfolio

https://doi.org/10.1038/s41586-025-09032-9

Supplementary information

Preparation of a neutral nitrogen allotrope hexanitrogen C_{2h}-N_6

In the format provided by the authors and unedited

Supplementary Information

Preparation of a Neutral Nitrogen Allotrope Hexanitrogen C_{2h}-N_6

Weiyu Qian (钱伟煜), Artur Mardyukov* & Peter R. Schreiner*

Institute for Organic Chemistry, Justus Liebig University, Giessen 35392 (Germany)

Table of Contents

Note for the Synthesis 2
Fig. S1 2
Fig. S2 3
Fig. S3 4
Fig. S4 5
Fig. S5 5
Fig. S6 6
Fig. S7 6
Fig. S8 7
Fig. S9 7
Fig. S10 8
Fig. S11 9
Fig. S12 9
Fig. S13 10
Fig. S14 10
Fig. S15 10
Table S1 11
Table S2 12
Table S3 13
Table S4 14
Coordinates (Ångstrom) and Energies (Hartree) 15

Note for the Synthesis

The gas-phase reaction is very sensitive to the apparatus setup. We have tried many configurations and succeeded with either a directly connected U-trap or a straight tube with a flying distance of approximately 15 mm from the front side of the quartz tube to the matrix window (Fig. S1). Quartz wool must not be used. The Young valves should be open completely when conducting reactions in a U-trap. The entire setup was covered by aluminium foil to avoid light exposure during the reactions.

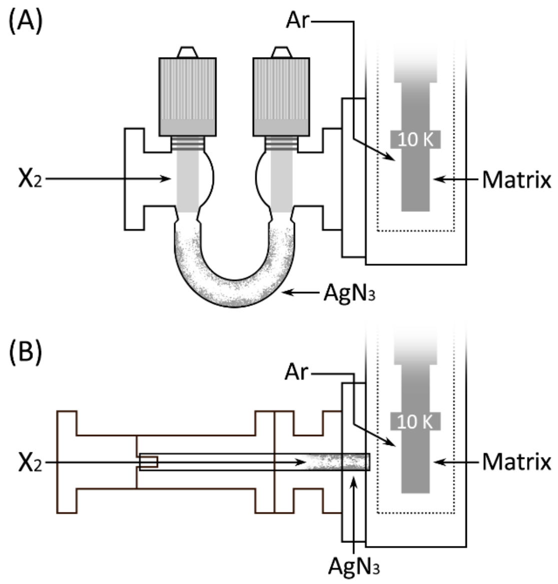

Fig. S1. Instrument setup: (A) U-trap; (B) quartz tube.

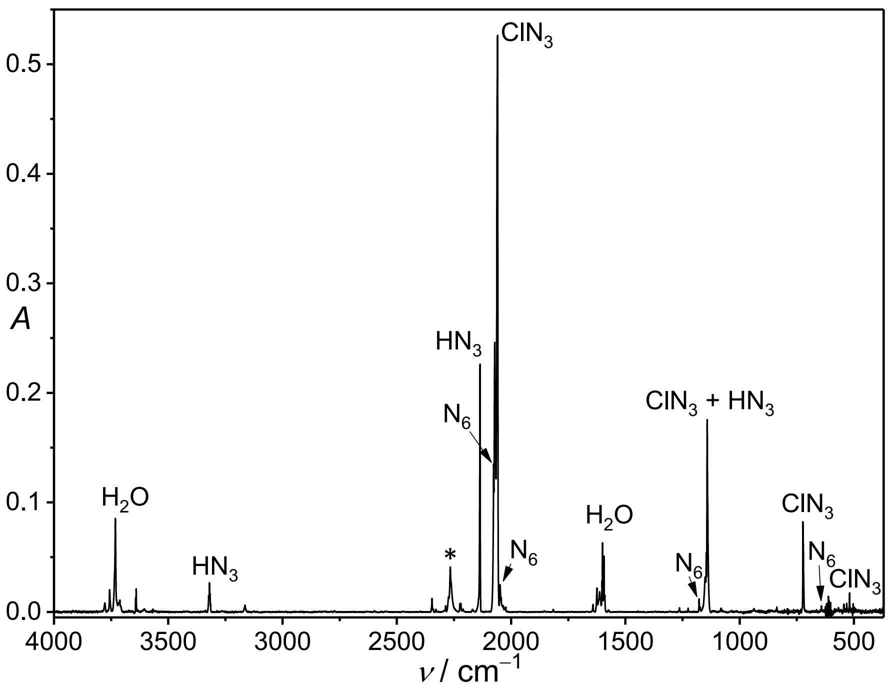

Fig. S2. Infrared spectrum of the deposition products of the reaction of Cl_2 and AgN_3.

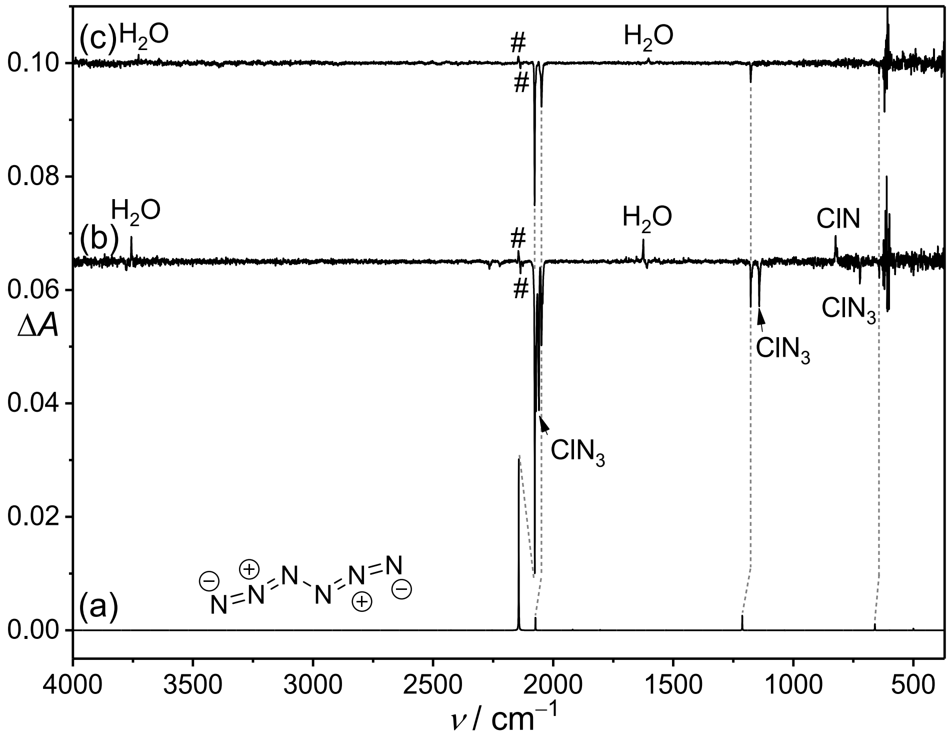

Fig. S3. Full-range version of Figure 1 in the main text. (a) Computed DVPT2 anharmonic infrared spectrum for C_{2h}-N_6 at B3LYP/def2-TZVP, including the combination ν_8 + ν_9. (b) Difference spectrum shows the changes after 8 min 436 nm irradiation of Cl_2 and AgN_3 reaction products. (c) Difference spectrum shows the changes after 6 min 436 nm irradiation of Br_2 and AgN_3 reaction products. Matrix sites from HN_3 (#) and H_2O are marked.

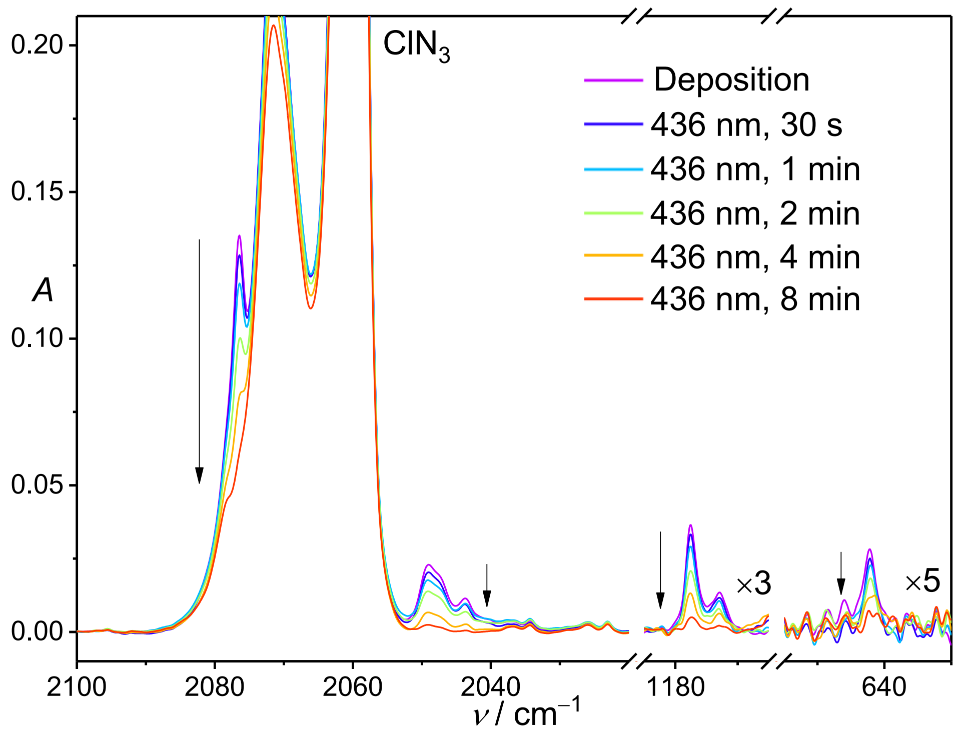

Fig. S4. Time-dependent infrared spectra upon 436 nm irradiation of Cl_2 and AgN_3 reaction products in an Ar-matrix (10 K).

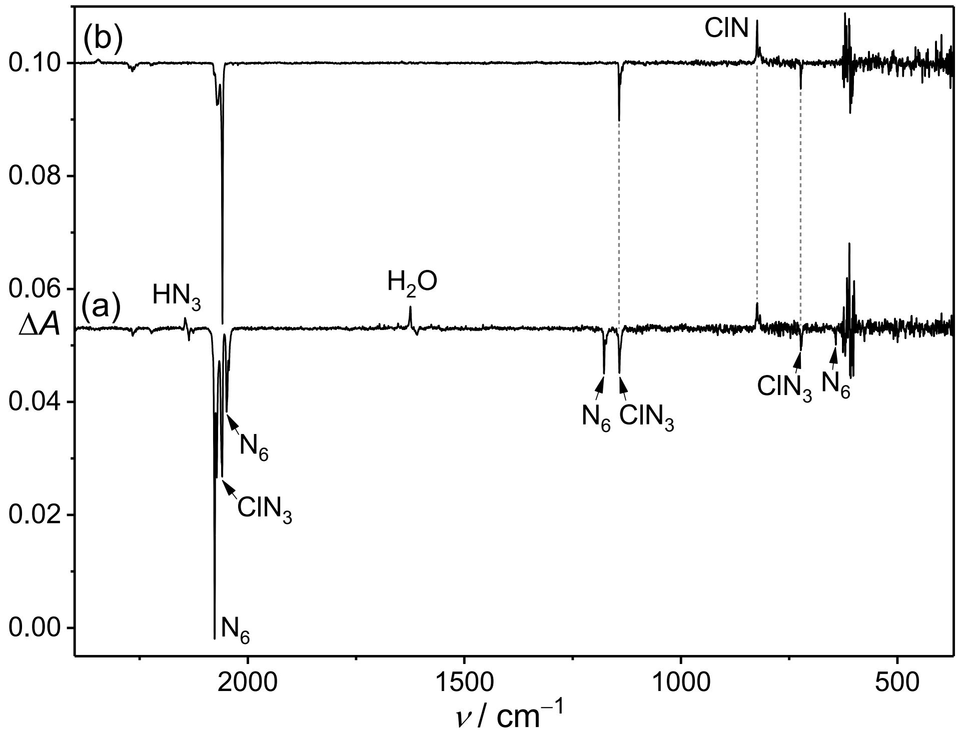

Fig. S5. (a) Difference spectrum showing the changes after 8 min 436 nm irradiation of Cl_2 and AgN_3 reaction products. (b) Difference spectrum showing the changes after 5 min 365 nm irradiation (peaks from N_6 had completely vanished in previous irradiation). Matrix sites from HN_3 (#) and H_2O are marked.

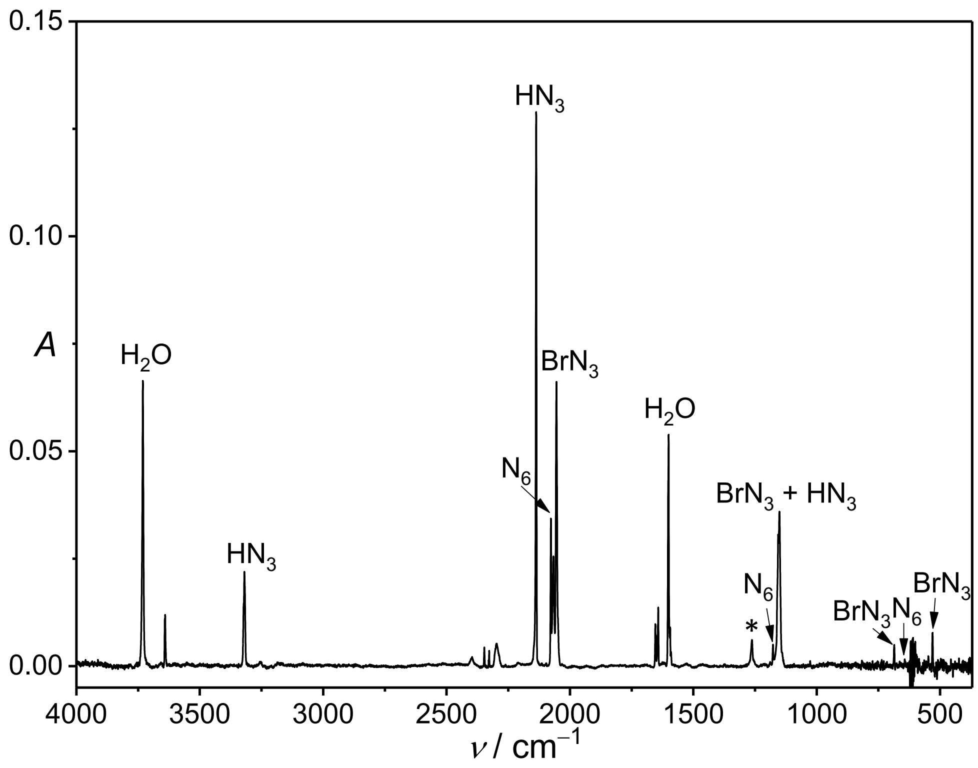

Fig. S6. Infrared spectrum of the deposition products of the reaction of Br_2 and AgN_3.

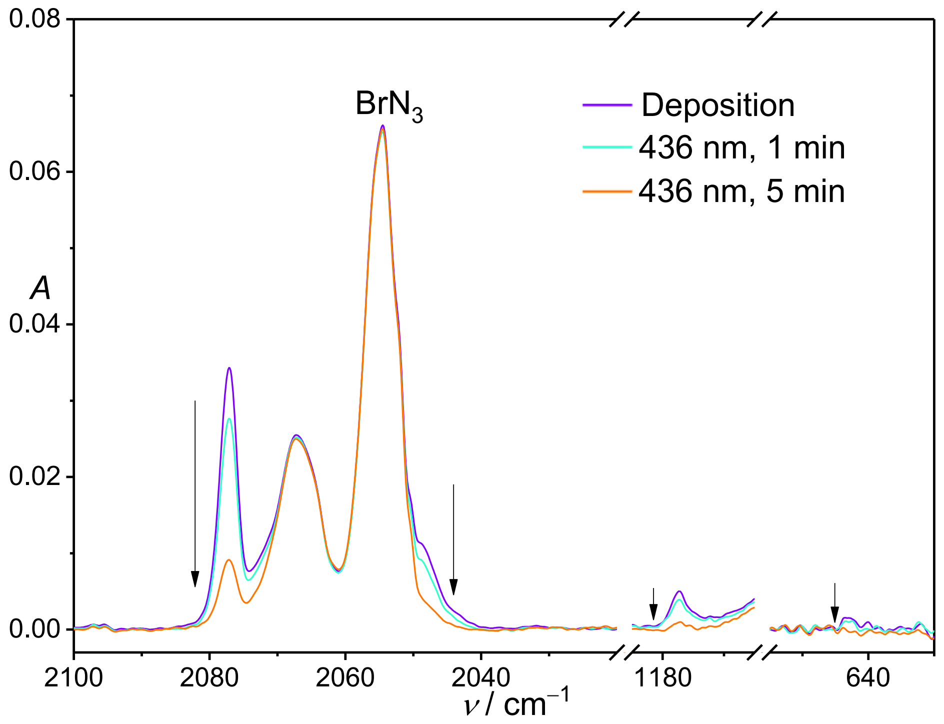

Fig. S7. Time-dependent infrared spectra upon 436 nm irradiation of Br_2 and AgN_3 reaction products in an Ar-matrix (10 K).

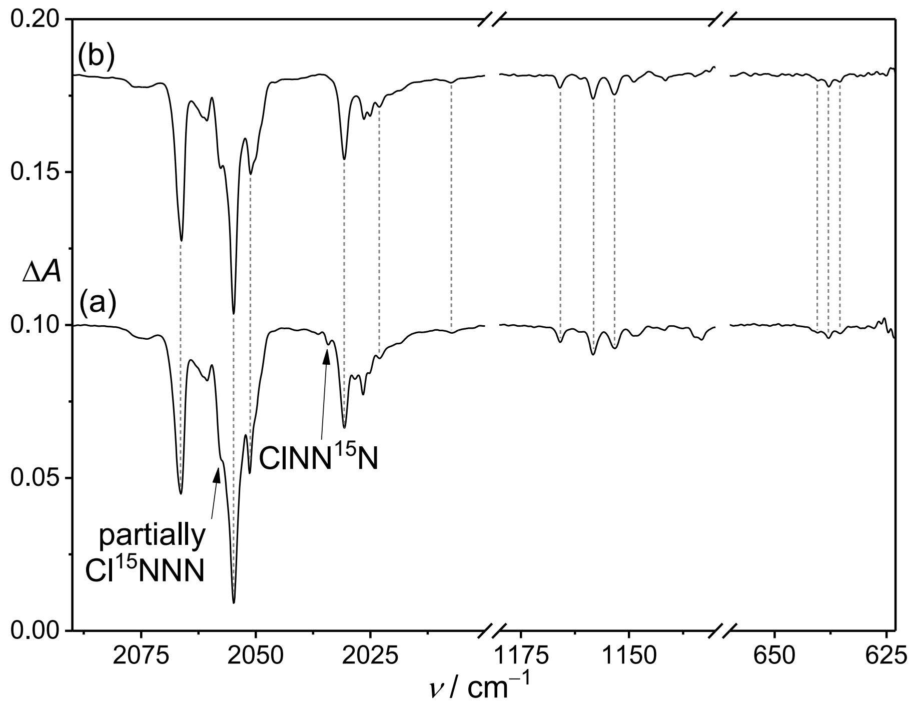

Fig. S8. (a) Difference spectrum showing the changes after 8 min 436 nm irradiation of Cl_2 and $AgNN^{15}N$ reaction products. (b) Difference spectrum shows the changes after 6 min 436 nm irradiation of Br_2 and $AgNN^{15}N$ reaction products.

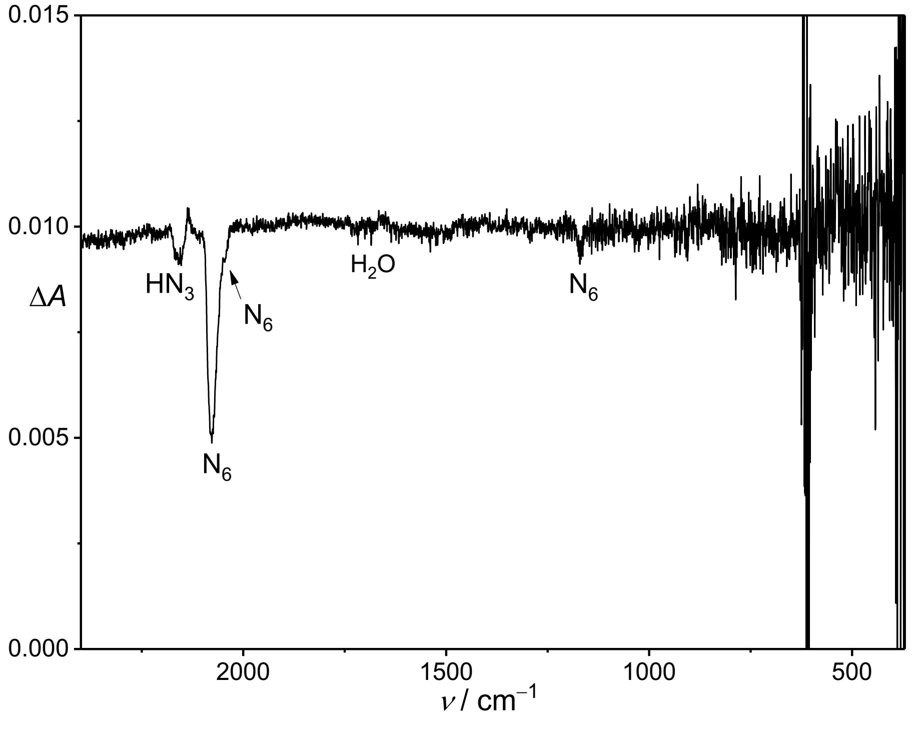

Fig. S9. Difference spectrum of C_{2h}-N_6 showing the changes after 8 min 436 nm irradiation of Br_2 and AgN_3 reaction products film at 77 K. Matrix sites from HN_3 and H_2O are marked.

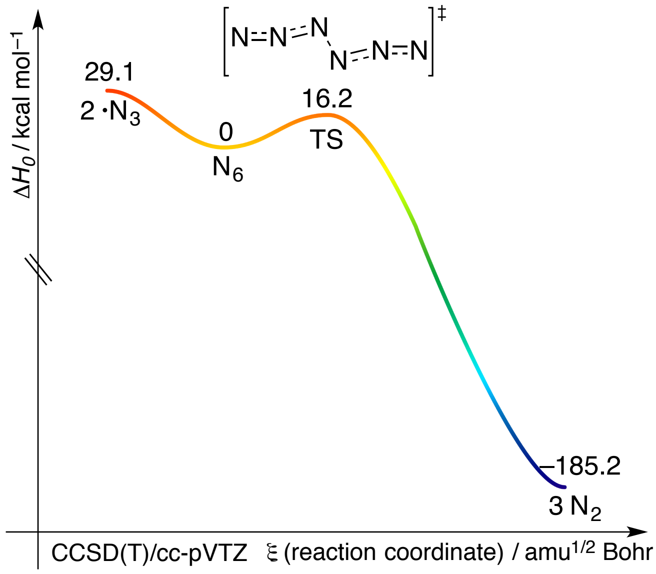

Fig. S10. Potential energy profile (ΔH_0, kcal mol^{-1}) for N_6 at CCSD(T)/cc-pVTZ + ZPVE.

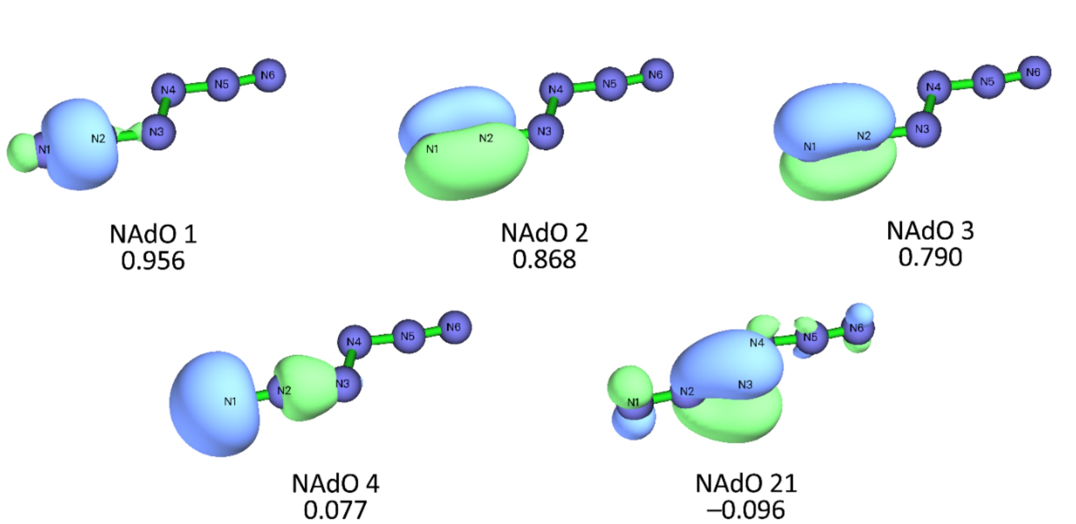

Fig. S11. Natural adaptive orbitals (NAdOs) of C_{2h}-N_6 from bond order density (BOD) analysis of bond N1-N2 (isovalue = 0.064), eigenvalues of NAdOs are given.

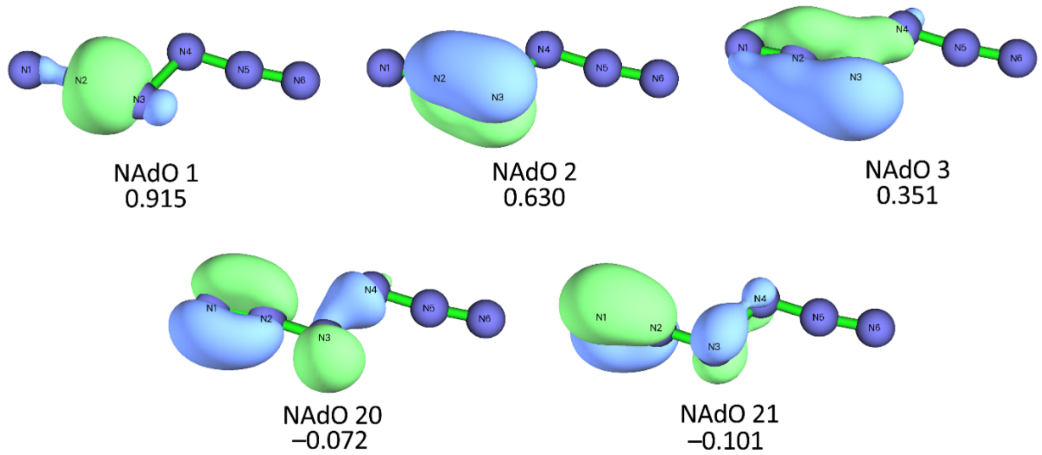

Fig. S12. Natural adaptive orbitals (NAdOs) of C_{2h}-N_6 from bond order density (BOD) analysis of bond N2-N3 (isovalue = 0.064), eigenvalues of NAdOs are given.

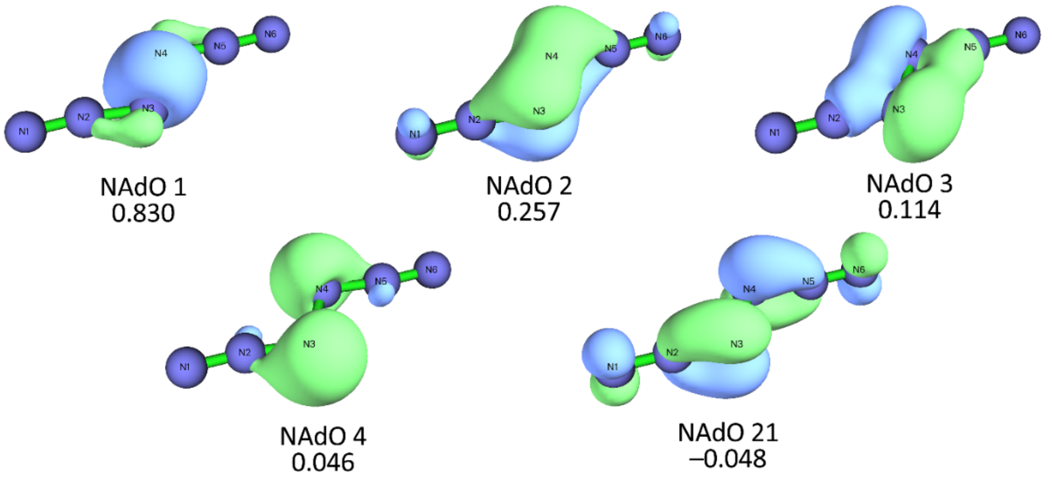

Fig. S13. Natural adaptive orbitals (NAdOs) of C_{2h}-N_6 from bond order density (BOD) analysis of bond N3-N4 (isovalue = 0.064), eigenvalues of NAdOs are given.

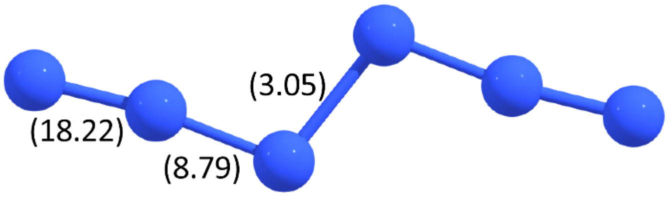

Fig. S14. The computed bond force constants (aJ Å^{-2}) of C_{2h}-N_6.

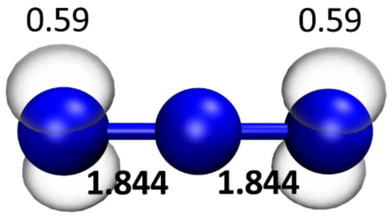

Fig. S15. Computed spin density of azide radical. Mulliken spin polulation (in regular) and natural bond order (in bold) are shown.

Table S1. Experimental and computed IR frequencies (> 400 cm^{-1}) and intensities (km mol^{-1}) for C_{2h}-N_6.

$\nu_{cal.}$		$\nu_{exp.}$	$\Delta\nu_{cal.}(^{15}N/^{14}N)$[c]			$\Delta\nu_{exp.}(^{15}N/^{14}N)$			assignment
B3LYP/DVPT2[a]	CCSD(T)/harm[b]	Ar-matrix	**1a**	**1b**	**1c**				
2198.1 (0)	2185.0 (0)	n.o.	24.5	11.6	10.0	n.o.	n.o.	n.o.	ν_{12}, A_g, ν_{sym} N1N2N3
2143.5 (1095)	2125.0 (1180)	2076.6 (s)	24.4	16.7	4.8	25.9	22.2	10.9	ν_{11}, B_u, ν_{asym} N1N2N3
2074.0 (80)[d]	2102.5	2049.0 (m)	12.2	29.5	44.0	17.6	27.8	41.2	$\nu_8 + \nu_9$
1259.6 (0)	1265.3 (0)	n.o.	8.1	19.3	30.2	n.o.	n.o.	n.o.	ν_{10}, A_g, ν_{sym} N3N2N1
1212.6 (95)	1202.4 (120)	1177.6 (m)	11.3	18.7	23.6	11.6	19.3	24.3	ν_9, B_u, ν_{asym} N3N2N1
869.1 (0)	900.0 (0)	871.4[e]	0.6	11.4	22.4	8.3[e]	8.5[e]	16.9[e]	ν_8, A_g, ν N3N4
660.5 (41)	648.5 (31)	642.1 (w)	1.5	4.0	6.5	1.4	4.3	7.3	ν_7, B_u, δ_{asym} N1N2N3
598.0 (0)	599.2 (0)	n.o.	5.7	4.4	4.1	n.o.	n.o.	n.o.	ν_6, A_g, δ_{sym} N1N2N3
537.0 (0)	531.4 (0)	n.o.	3.7	2.6	2.5	n.o.	n.o.	n.o.	ν_5, B_g, ω_{sym} N1N2N3
500.2 (11)	496.0 (10)	n.o.	3.4	2.8	3.1	n.o.	n.o.	n.o.	ν_4, A_u, ω_{asym} N1N2N3

[a] Anharmonic frequencies computed at B3LYP/def2-TZVP using the DVPT2 method.

[b] Unscaled harmonic frequencies computed at CCSD(T)/cc-pVTZ.

[c] Anharmonic isotope shifts computed at B3LYP/def2-TZVP using the DVPT2 method.

[d] B3LYP/def2-TZVP computation suggests there is Fermi reson~~n~~ance with ν_{11}.

[e] Derived from a combination band.

Table S2. Experimental and computed IR frequencies (> 400 cm^{-1}) and intensities (km mol^{-1}) with different methods for C_{2h}-N_6.

$\nu_{cal.}$			$\nu_{exp.}$	assignment
B3LYP/harm[a]	B3LYP/DVPT2[b]	CCSD(T)/harm[c]	Ar-matrix	
2249.5 (0)	2198.1 (0)	2185.0 (0)	n.o.	ν_{12}, A_g, ν_{sym} N1N2N3
2191.7 (1314)	2143.5 (1095)	2125.0 (1180)	2076.6 (s)	ν_{11}, B_u, ν_{asym} N1N2N3
2145.1	2074.0 (80)	2102.5	2049.0 (m)	$\nu_8 + \nu_9$
1306.1 (0)	1259.6 (0)	1265.3 (0)	n.o.	ν_{10}, A_g, ν_{sym} N3N2N1
1251.5 (121)	1212.6 (95)	1202.4 (120)	1177.6 (m)	ν_9, B_u, ν_{asym} N3N2N1
893.6 (0)	869.1 (0)	900.0 (0)	871.4[d]	ν_8, A_g, ν N3N4
665.7 (42)	660.5 (41)	648.5 (31)	642.1 (w)	ν_7, B_u, δ_{asym} N1N2N3
611.1 (0)	598.0 (0)	599.2 (0)	n.o.	ν_6, A_g, δ_{sym} N1N2N3
545.0(0)	537.0 (0)	531.4 (0)	n.o.	ν_5, B_g, ω_{sym} N1N2N3
510.6 (12)	500.2 (11)	496.0 (10)	n.o.	ν_4, A_u, ω_{asym} N1N2N3

[a]Unscaled harmonic frequencies computed at B3LYP/def2-TZVP.

[b]Anharmonic frequencies computed at B3LYP/def2-TZVP using the DVPT2 method.

[c]Unscaled harmonic frequencies computed at CCSD(T)/cc-pVTZ.

[d]Derived from a combination band.

Table S3. Computed anharmonic IR frequencies (DVPT2 method at B3LYP/def2-TZVP, > 400 cm^{-1}) and intensities (km mol^{-1}) for isotopomers of C_{2h}-N_6.

$\nu_{cal.}$				Mode
natural	^{15}NNNNN^{15}N	^{15}NNN^{15}NNN	NN^{15}N^{15}NNN	
2198.1 (0)	2173.6 (0)	2186.5 (13)	2193.6 (0)	ν_{12}
2143.5 (1095)	2119.1 (1041)	2126.8 (1068)	2138.7 (1125)	ν_{11}
2074.0 (80)	2061.7 (128)	2044.4 (81)	2030.0 (35)	$\nu_8 + \nu_9$
1259.6 (0)	1251.5 (0)	1240.4 (5)	1229.4 (0)	ν_{10}
1212.6 (95)	1201.3 (92)	1193.9 (98)	1189.1 (107)	ν_9
869.1 (0)	868.6 (0)	857.8 (< 1)	846.7 (0)	ν_8
660.5 (41)	659.0 (41)	656.5 (40)	654.0 (40)	ν_7
598.0 (0)	592.3 (0)	593.5 (< 1)	594.8 (0)	ν_6
537.0 (0)	533.2 (0)	534.3 (< 1)	535.4 (0)	ν_5
500.2 (11)	496.6 (11)	497.2 (11)	498.0 (11)	ν_4

Table S4. Computed rate constants (s^{-1}) for the decomposition of C_{2h}-N_6 with different models included in Polyrate17C at the B3LYP/def2-TZVP level at different temperature.

T/K	TST	CVT	CVT/ZCT	CVT/SCT
2.8				
10.0				
20.0	9.09E-171	2.08E-171	5.06E-19	1.68E-14
28.0	1.12E-118	3.85E-119	3.63E-18	4.06E-14
40.0	1.47E-79	6.69E-80	1.82E-16	5.01E-13
50.0	2.81E-61	1.44E-61	2.27E-15	3.21E-12
75.0	7.72E-37	4.48E-37	2.37E-13	1.23E-10
77.3	2.40E-35	1.40E-35	3.50E-13	1.66E-10
100.0	1.46E-24	8.71E-25	1.36E-11	2.56E-09
125.0	3.72E-17	2.20E-17	8.94E-10	4.98E-08
150.0	3.45E-12	1.99E-12	6.29E-08	1.01E-06
175.0	1.28E-08	7.15E-09	3.72E-06	2.09E-05
194.7	1.92E-06	1.05E-06	7.85E-05	2.32E-04
200.0	6.29E-06	3.41E-06	1.73E-04	4.46E-04
225.0	8.04E-04	4.20E-04	5.86E-03	9.65E-03
250.0	3.98E-02	2.00E-02	1.31E-01	1.75E-01
273.1	7.95E-01	3.86E-01	1.61E+00	1.96E+00
275.0	9.88E-01	4.79E-01	1.94E+00	2.35E+00
298.1	1.21E+01	5.66E+00	1.69E+01	1.94E+01

Coordinates (Ångstrom) and Energies (Hartree)

***C_{2h}*-N_6**

B3LYP/Def2-TZVP

N	0.12299000	-1.62281000	0.00000000
N	-0.55232000	0.46173000	0.00000000
N	-0.12299000	1.62281000	0.00000000
N	-0.12299000	-2.72347000	0.00000000
N	0.12299000	2.72347000	0.00000000
N	0.55232000	-0.46173000	0.00000000

Zero-point correction= 0.024497

Thermal correction to Energy= 0.029944

Thermal correction to Enthalpy= 0.030888

Thermal correction to Gibbs Free Energy= -0.004602

Sum of electronic and zero-point Energies= -328.437429

Sum of electronic and thermal Energies= -328.431982

Sum of electronic and thermal Enthalpies= -328.431038

Sum of electronic and thermal Free Energies= -328.466528

***C_{2h}*-N_6**

CCSD(T)/cc-pVTZ (internal coordinates)

N	0	0	0	0.000000000000	0.00000000	0.00000000
N	1	0	0	2.183250347540	0.00000000	0.00000000
N	2	1	0	1.251470563090	140.26056724	0.00000000
N	1	2	3	1.138174076326	147.74187273	179.99999791
N	3	2	1	1.138173914125	172.51870278	180.00000121
N	1	2	3	1.251470292364	39.73940168	0.00000000

CCSD(T)/cc-pVTZ electronic energy= -327.83100190

CCSD(T)/cc-pVTZ enthalpy= -327.80072255

ZPVE (CCSD(T)/cc-pVTZ) = 0.02376194

CCSD(T)/cc-pVTZ free energy= -327.83552055

N_3 radical (for bond dissociation energy, optimized)

CCSD(T)/cc-pVTZ (internal coordinates)

N	0	0	0	0.000000000000	0.00000000	0.00000000
N	1	0	0	1.183096393309	0.00000000	0.00000000
N	2	1	0	1.183097047958	179.99201228	0.00000000

CCSD(T)/cc-pVTZ electronic energy= -163.88936302

CCSD(T)/cc-pVTZ enthalpy= -163.87719650

ZPVE (CCSD(T)/cc-pVTZ) = 0.00815819

CCSD(T)/cc-pVTZ free energy= -163.89696460

N_2

CCSD(T)/cc-pVTZ (internal coordinates)

N	0	0	0	0.000000000000	0.00000000	0.00000000
N	1	0	0	1.103764945364	0.00000000	0.00000000

CCSD(T)/cc-pVTZ electronic energy= -109.37393684

CCSD(T)/cc-pVTZ enthalpy= -109.36528827

ZPVE (CCSD(T)/cc-pVTZ) = 0.00534378

CCSD(T)/cc-pVTZ free energy= -109.38704037

TS for dissociation into 3 N_2

B3LYP/Def2-TZVP

N	-1.65648200	-0.18493100	0.04565900
N	0.50636600	0.53652100	0.40348600
N	1.65642700	-0.18484500	-0.04558600
N	-2.75997100	-0.35164900	0.04658100
N	2.75990400	-0.35171800	-0.04668600
N	-0.50624400	0.53662100	-0.40345400

Zero-point correction= 0.019154

Thermal correction to Energy= 0.025313

Thermal correction to Enthalpy= 0.026258

Thermal correction to Gibbs Free Energy= -0.011009

Sum of electronic and zero-point Energies= -328.410897

Sum of electronic and thermal Energies= -328.404737

Sum of electronic and thermal Enthalpies= -328.403793

Sum of electronic and thermal Free Energies= -328.441059

Imaginary frequency = 1064.2i cm^{-1}

HN_3

CCSD(T)/cc-pVTZ (internal coordinates)

N	0	0	0	0.000000000000	0.00000000	0.00000000
H	1	0	0	1.018084387995	0.00000000	0.00000000
N	1	2	0	1.247654099142	108.30841720	0.00000000
N	3	1	2	1.136185843383	171.65256383	179.94322536

CCSD(T) energy = -164.538676947267

N_2H_4

CCSD(T)/cc-pVTZ (internal coordinates)

N	0	0	0	0.000000000000	0.00000000	0.00000000
H	1	0	0	1.015363736070	0.00000000	0.00000000
H	1	2	0	1.011963693700	106.58076088	0.00000000
N	1	2	3	1.444844072999	110.78254425	244.88663846
H	4	1	2	1.011963733466	106.20306068	270.12547224
H	4	1	2	1.015363973025	110.78242868	25.47448396

CCSD(T) energy = -111.698718776961

***trans*-HNNH**

CCSD(T)/cc-pVTZ (internal coordinates)

N	0	0	0	0.000000000000	0.00000000	0.00000000
H	1	0	0	1.030943064633	0.00000000	0.00000000
N	1	2	0	1.253583342219	105.72863279	0.00000000
H	3	1	2	1.030943064632	105.72863280	180.00000000

CCSD(T) energy = -110.478027131268

TS for dissociation into 3 N_2

CCSD(T)/cc-pVTZ (internal coordinates)

N	0	0	0	0.000000000000	0.00000000	0.00000000
N	1	0	0	2.241861029835	0.00000000	0.00000000
N	2	1	0	1.569830117585	114.04432242	0.00000000
N	1	2	3	1.126410511898	161.64725208	105.40114534
N	3	2	1	1.118386052358	152.04470148	194.40036918
N	2	1	3	1.339279152996	33.55891178	91.74769990

CCSD(T)/cc-pVTZ electronic energy= -327.80072402

CCSD(T)/cc-pVTZ enthalpy= -327.77487147

ZPVE (CCSD(T)/cc-pVTZ) = 0.01873790

CCSD(T)/cc-pVTZ free energy= -327.81200831

Imaginary frequency = 966.3i cm^{-1}
